# Identifying and validating housekeeping hybrid *Prunus* spp. genes for root gene-expression studies

**DOI:** 10.1371/journal.pone.0228403

**Published:** 2020-03-18

**Authors:** Adriana Bastias, Kristen Oviedo, Ruben Almada, Francisco Correa, Boris Sagredo

**Affiliations:** 1 Facultad de Ciencias de la Salud, Instituto de Ciencias de la Salud, Universidad Autónoma de Chile, Avenida Pedro de Valdivia, Santiago, Chile; 2 Instituto de Investigaciones Agropecuarias (INIA) CRI Rayentué, Sector Los Choapinos, Rengo, Chile; 3 Centro de Estudios Avanzados en Fruticultura (CEAF), Sector Los Choapinos, Rengo, Chile; Nazarbayev University, KAZAKHSTAN

## Abstract

*Prunus* rootstock belonging to subgenera *Amygdalus* (peach), *Prunus* (plum) and *Cerasus* (cherry) are either from the same species as the scion or another one. The number of inter-species (including inter-subgenera) hybrids has increased as a result of broadening the genetic basis for stress (biotic and abiotic) resistance/tolerance. Identifying genes associated with important traits and responses requires expression analysis. Relative quantification is the simplest and most popular alternative, which requires reference genes (housekeeping) to normalize RT-qPCR data. However, there is a scarcity of validated housekeeping genes for hybrid *Prunus* rootstock species. This research aims to increase the number of housekeeping genes suitable for *Prunus* rootstock expression analysis.

Twenty-one candidate housekeeping genes were pre-selected from previous RNAseq data that compared the response of root transcriptomes of two rootstocks subgenera to hypoxia treatment, ‘Mariana 2624’ (*P*. *cerasifera* Ehrh.× *P*. *munsoniana* W. Wight & Hedrick), and ‘Mazzard F12/1’ (*P*. *avium* L.). Representing groups of low, intermediate or high levels of expression, the genes were assayed by RT-qPCR at 72 hours of hypoxia treatment and analyzed with NormFinder software. A sub-set of seven housekeeping genes that presented the highest level of stability were selected, two with low levels of expression (*Unknown 3*, *Unknown 7*) and five with medium levels (*GTB 1*, *TUA 3*, *ATPase P*, *PRT 6*, *RP II*). The stability of these genes was evaluated under different stress conditions, cold and heat with the hybrid ‘Mariana 2624’ and N nutrition with the hybrids ‘Colt’ (*P*. *avium* × *P*. *pseudocerasus* Lindl.) and ‘Garnem’ **[***P*. *dulcis* Mill.× (*P*. *persica* L.× *P*. *davidiana* Carr.)**]**. The algorithms of geNorm and BestKeeper software also were used to analyze the performance of these genes as housekeepers.

Stability rankings varied according to treatments, genotypes and the software for evaluation, but the gene *GBT 1* often had the highest ranking. However, most of the genes are suitable depending on the stressor and/or genotype to be evaluated. No optimal number of reference genes could be determined with geNorm software when all conditions and genotypes were considered. These results strongly suggest that relative RT-qPCR should be analyzed separately with their respective best housekeeper according to the treatment and/or genotypes in *Prunus* spp. rootstocks.

## Introduction

Stone fruit trees (*Prunus* spp.) are economically important because they produce edible fruits such as peaches, cherries, and apricots. Most stone fruit trees are grafted on rootstocks (seedlings or clonally propagated) that belong to either the same or other *Prunus* species [1]. Therefore, these fruit trees are composed of two parts, the rootstock and scion. Rootstocks are responsible for absorbing water and nutrients and providing resistance to soil pathogens, pests and tolerance to environmental conditions. Adaptation to environmental stressors like drought, salinity, cold, and root hypoxia are largely determined by the rootstock [2]. Other important attributes of fruit cultivars like initial flowering, vigor, nutritional state, fruit production, size, and taste can be significantly influenced by the rootstock [3,4]. To be grafted, rootstocks must be compatible with a wide range of cultivars, resistant to pests and diseases, and suited to different soil types [5]. It is unlikely that any single *Prunus* genotype has all these attributes. Incorporating the maximum number of these characteristics to increase usefulness and areas of adaptation is the main goal of *Prunus* rootstock breeding programs [6]. However, there are important limitations to traditional breeding programs, including long generation times and extensive space requirements. Deepening our understanding of the molecular basis of the traits/responses of *Prunus* rootstocks and identifying the genes involved will be critical steps toward improving rootstock plants by marker-assisted selection (MAS) methods [7]. Other approaches are also possible, based on direct modification of key genes associated with traits of interest by genetically engineering *Prunus* species [8]. However, the molecular basis of *Prunus* rootstock traits and responses remain largely unknown. Gene expression studies using NGS (Next Generation Sequencing) and/or candidate genes from other model plants represent valuable approaches to identifying key candidate genes underlying traits of interest [9].

Quantitative reverse transcription PCR (RT-qPCR) is a powerful tool for quantifying gene expression due to its high degree of sensitivity, specificity and reproducibility [10]. Normalizing RT-qPCR data is crucial to obtain results as close to reality as possible. Biological (gene-specific) and technical (RNA quantity and quality, RT efficiency and PCR efficiency) variations occur in gene expression analysis. Appropriate normalization strategies are required to control experimental error during the multistage process, which also include extracting and processing RNA samples [11]. There are several qPCR methods of absolute and relative quantification. Absolute quantification, which require normalizing to sample size or a standard curve, quantifies transcript in a given sample [12]. Relative quantification analyzes changes in gene expression in a given sample relative to a reference sample. Among several proposed methods, reference genes, also named control or housekeeping genes, are frequently used to normalize RT-qPCR data [11,12]. Housekeeping genes function as internal controls that are subject to the same conditions as the mRNAs of interest, which are also measured by real time RT-qPCR [11]. A serious limitation of this type of gene expression study of *Prunus* rootstocks is the scarcity of validated reference genes. Because modern *Prunus* rootstocks include genotypes of different peach, plum and cherry subgenera, including several inter-specific hybrids, identifying reference genes with stable expression among *Prunus* species is a challenge.

Several plant genes have been used as internal controls in expression studies, such as *glyceraldehyde-3-phosphate dehydrogenase* (*GAPDH*), *tubulin* (*TUB*), *actin* (*ACT*) and *18S ribosomal RNA* (*18S rRNA*) [13,14]. However, there are no universal reference genes with constant levels of expression for all plants, tissue, treatments and developmental stages. Stable expression in one organism or species may not be suitable for standardization in another, or for the same tissue but under different conditions [13]. Hence, researcher must determine the best housekeeping genes according to the specific experimental conditions. Identifying these genes is not a simple process. It consists of two steps: first, identifying likely candidate genes and then determining their stability [15].

Different algorithms have been developed to determine reliability of RT-qPCR results in gene expression, which differ on the approaches used to account for non-biological variations. The three more widely applied algorithms are NormFinder, geNorm and BestKeeper [15–17]. NormFinder measures variation in function of variance and ranks putative housekeeping genes according to how they differ among and within groups, while avoiding co-regulated reference genes. BestKeeper and geNorm both use the geometric mean, but BestKeeper uses raw data, and analyses up to 10 references genes, while geNorm determines stability M-value using the average pairwise variation of each candidate gene [15–17].

In this work, we searched for and validated housekeeping genes for *Prunus* rootstock gene expression analysis. We assessed candidate housekeeping genes from our existing RNAseq data [18] and in the literature [19]. The RT-qPCR data were analyzed using algorithms NormFinder, geNorm and BestKeeper [15–17], to determine suitable *Prunus* spp. housekeeping genes for six experimental conditions: hypoxia, drought, salinity, cold, heat and N nutrition.

## Materials and methods

### Plant material

Clonally propagated and virus-free rootstock plants from ‘Mariana 2624’ (*P*. *cerasifera* × *P*. *munsoniana* W. Wight & Hedrick), ‘Mazzard F12/1’ (*P*. *avium*), ‘Colt’ (*P*. *avium* (L.) L. × *P*. *pseudocerasus* Lindl.) and ‘Garnem’ [*P*. *dulcis* × (*P*. *persica* × *P*. *davidiana*)] were donated by a commercial nursery (Agromillora Sur, S.A., Curicó, Chile). Plants were transplanted to 2-L plastic pots with a mixture of vermiculite:perlite:sand (1:1:1v/v) as a substrate. The plants were maintained in the field under a shade net (Raschel sun shading net with 50% shading) at the Instituto de Investigaciones Agropecuarias–Rayentué,Rengo, Chile (latitude 34°19'16.1"S and longitude 70°50'03.6"W.) during two growing seasons (2013–2014 and 2014–2015) until they were used in different experiments. Before treatments, the plants were regularly watered three times a week with 200 ml of tap water and fertilized every 2 weeks with 1 g/pot with N:P:K (25:10:10) (Ultrasol^™^, Soquimich, Chile).

### Abiotic stress treatments

Abiotic stress treatments were performed during the first two weeks of December 2014 with a photoperiod 14.2 h day length. Details of climatic conditions can be consulted at Rengo agroclimatic station of INIA-Agromet (http://agromet.inia.cl).

Hypoxia experiments as described by Arismendi et al. [18] were carried out. The plants were maintained in the field under a shade net (Raschel sun shading net with 50% shading) during the hypoxia experiments. With the exception of the control plants, the plants in their pots were placed in 100-L plastic containers when they reached an average height of 30 cm. Root hypoxia was generated by filling the plastic containers with water until approximately 4 cm above the level of the pot substrate, and then bubbling 100% gaseous N_2_ (1 L/min) through the water to displace dissolved O_2_. The oxygen levels in close proximity to the plant roots were monitored throughout the experiment with an oxygen-electrode (Extech Instrument, MA, USA) until to reaching ≤ 4 mg/L. With a total of six plants per genotype, roots from three randomly selected plants were collected at 0 and 72 h. Samples collected at time 0 h represented the control plants (without flooding). To take root samplings, the soil was completely removed and the plant roots were gently washed with tap water, then excised from the plants, immediately frozen in liquid nitrogen, and stored at −80°C until RNA extraction.

Saline, cold, heat and drought stress treatments were performed as described by [20,21]. A total of 27 plants ‘Mariana 2624’ plants of uniform size (30 cm high) were used for these stress experiments. For saline treatment, the roots of six plants were immersed in a 200 mM NaCl solution. For the cold and heat treatments, six plants each were kept in growth chambers at respectively 4 and 37°C. For the drought treatment, roots from six plants were washed gently with water to remove soil and then put in perlite for rapid dehydration [21]. Roots from three plants were collected at 0, 6 and 24 h of their respective stress treatment. Time 0 h represent the control for all stress treatments. The removed roots were immediately frozen in liquid nitrogen and stored at −80°C for RNA extraction and gene expression analysis.

### Nitrogen (N) treatment

A total of nine plants per genotype, ‘Garnem’ [*P*. *dulcis* × (*P*. *persica* × *P*. *davidiana*)] and ‘Colt’ (*P*. *avium* × *P*. *pseudocerasus*), were transplanted to 2-L plastic pots with a mixture of vermiculite:perlite (1:1 v/v) as substrate. Plants were maintained in the field under a shade net (Raschel sun shading net with 50 shading) at the Instituto de Investigaciones Agropecuarias—Rayentué (Rengo, Chile) during two consecutive growing seasons (2013–2014 and 2014–2015). Plants were watered twice a week, once with tap water and the other with a modified Murashige & Skoog nitrogen-free basal medium (Phytotechnology Laboratories, M531), supplemented with 200 ml of 0.0, 0.1 and 10.0 mM of ammonium nitrate (NH_4_NO_3_), for two consecutive months of plant growth during two seasons. Low and high rate of fertilization of nitrogen were represented by 0.1 mM and 10.0 mM of ammonium nitrate [22]. The roots of three plants of each genotype x N-treatment were sampled at the end of the second season (April 2015). Control plants with 0.0 mM of supplementary ammonium nitrate were maintained under standard irrigation conditions, with watering twice a week with 200 ml of deionized water, and fertilizing every two weeks with 1 g/pot of N:P:K (25:10:10) (Ultrasol^™^, Soquimich, Chile). For root samplings, the soil was completely removed and plant roots were gently washed with tap water, excised from the plants, immediately frozen in liquid nitrogen and stored at −80°C until RNA extraction.

### Gene expression analysis by RT-qPCR

Total RNA of three plant per treatment and three control plants were used for all gene expression analysis by quantitative PCR (RT-qPCR). Total RNA was extracted from root samples of control and treated plants according to Chang et al. [23]. Following the DNase I (RNase-Free, Ambion, Inc. Applied Biosystems) treatment, 5 μg of total RNA was used to synthesize cDNA from each sample, using a Thermoscript RT-PCR System^™^ (Invitrogen, Inc., Carlsbad, CA) with oligo(dT) primers. Gene transcript levels were measured by RT-qPCR using a Mx3000P QPCR System (Agilent Technologies, Santa Clara, CA). All reactions were made with the Brilliant SYBR Green Master Mix (Stratagene Inc., Santa Clara, CA), according to the manufacturer’s instructions. All RT-qPCR reactions were done in triplicate (technical replicates) using 2 μL Master Mix, 0.5 μL 250 nM of each primer, 1 μL of diluted cDNA and nuclease-free water to a final volume of 20 μL. Controls (with no cDNA and RNA without RT) were included in all runs. Fluorescence was measured at the end of the amplification cycles (Ct). Amplification was followed by melting curve analysis with continual fluorescence data acquisition from 65 to 95°C.

### Selection of *Prunus* putative housekeeping genes

Stable candidate genes were selected from a previous analysis of RNA sequencing of *Prunus* rootstocks [18] to be evaluated as expressed reference genes in RT-qPCR studies. Several classical reference genes described by Tong et al. [19] were also included. The previous RNA sequencing data consisted of root transcriptomes from two rootstocks genotypes, ‘Mariana 2426’ and ‘Mazzard F12/1’, with 0, 6, 24 and 72 h of hypoxia treatment [18]. Briefly, gene abundance was estimated by FPKM count (fragments per kilobase of transcript per million mapped reads) [24,25]. As a cutoff, we only considered genes with a minimum of 10 aligned fragments (-c option). To consider a gene as a candidate housekeeper, the calculated fold change among all time points had to be between -0.3 to +0.3. The coefficient variance (cv) values less than 0.3 and FDR-corrected P values <0.05 were used as filters. Categories of levels of expression were defined to ensure representability low (reads < 200), intermediate (200< reads < 3000) and high levels of expression (reads > 3,000). Primers for genes suitable for RT-qPCR were designed with Primer Premier 6.0 software (Premier Biosoft Interpairs, Palo Alto, CA), with a melting temperature between 58–61°C, 21–23 bp and approximately 50% GC content. Amplicon lengths were between 160–280 bp. All primers were synthetized at IDT (Integrated DNA Technologies, Inc., CA).

### Determining stability of housekeeping gene expression and statistical analysis

The expression levels of the candidate reference genes were determined by the number of amplification cycles (Cq) needed to reach a specific threshold level of detection. The obtained data were analyzed using NormFinder [15], geNorm [16] and BestKeeper software packages [17]. When the BestKeeper was used, the standard deviation (SD), coefficient of variation (CV) and pair of correlation coefficients (Poisson correlation coefficient) were calculated [16].

RT-qPCR data were exported to an Excel datasheet, and the Ct values were converted according to software requirements. Each of these approaches generates a measure of housekeeping gene stability, which can be used to rank genes as candidate housekeeping genes according to their stability.

## Results

### Identification of putative housekeeping genes for *Prunus* spp.

A total of 21 genes were selected for this study to identify housekeeping genes with different levels of expression for RT-qPCR studies of *Prunus* spp. (Table 1). Previous data analysis of RNA sequencing of root transcriptomes of two *Prunus* spp. rootstocks assayed under hypoxia [18] were used to select putative reference genes for *Prunus* spp. root expression. The genotypes ‘Mariana 2624’ and ‘Mazzard F12/1’, which are tolerant and sensitive to hypoxia, belong to different subgenera *Prunus* and *Cerasus*, respectively. Using RNAseq data from Arismendi et al. [18], most stable genes among treatments and genotypes were identified. With levels of expression relatively constant (fold changes between -0.3 and +0.3 and CV <0.3) a total of 611 candidate housekeeping genes were identified (S1 Table). Then, a set of twenty-one genes were selected to be evaluated by RT-qPCR, with 4, 10 and 7 of them respectively representing low (reads<200), intermediate (200>reads>3,000) and high levels expression (reads>3,000) (Table 1). This range of gene expression was chosen to identify suitable reference genes for better relative quantification of genes of interest with low, intermediate and high levels of expression. They comprise both widely-used classical reference genes like *GAPDH*, *TUB* and *ACT* [19], and ones less commonly used like a cyclophilin-like peptidyl-prolyl cis-trans isomerase family protein-coding genes (*CYP2*), a global transcription factor of group B1 (*GTB1*) and a Got1/Sft2-like vesicle transport protein (*GOT 1*) (Table 1).

**Table 1:** Candidate housekeeping genes selected in this study.

Name	*P*. *persica* database accession number	RNAseq Expression level	Arabidopsis homolog *locus*	*A*. *thaliana locus* description	Protein similarity (%)
*Unknown 2*	ppa013569	Low	unknown	Unknown	-
*Unknown 3*	ppb022375	Low	unknown	Unknown	-
*Got 1*	ppa013371	Low	AT5G24170	Got1/Sft2-like vesicle transport protein family	72.8
*Unknown 7*	ppa026944	Low	unknown	Unknown	-
*CYP 2*	ppa011090	Medium	AT4G34960	Cyclophilin-like peptidyl-prolyl cis-trans isomerase protein	86.2
*RP II*	ppa008812	Medium	AT2G15430	DNA-directed RNA polymerase family protein	92.8
*3B*	ppa000339	Medium	AT5G64270	Splicing factor. Putative	89.7
*ATPase P*	ppa000424	Medium	AT5G23630	Phosphate deficiency response 2	84.8
*PRT 6*	ppa000069	Medium	AT5G02310	Proteolysis 6	60.9
*VPS 13*	ppa000004	Medium	AT4G17140	Pleckstrin homology (PH) domain-containing protein	76.2
*PHD*	ppa000413	Medium	AT3G02890	RING/FYVE/PHD zinc finger superfamily protein	33.1
*LBA 1*	ppa000334	Medium	AT5G47010	RNA helicase. Putative	82.8
*GTB 1*	ppa000164	Medium	AT1G65440	global transcription factor group B1	68.3
*TUA 3*	ppa005642	Medium	AT1G50010	tubulin alpha-2 chain	93.6
*TUB*	ppa005644	High	AT1G20010	tubulin beta-5 chain	94.7
*GAPDH*	ppa008250	High	AT3G04120	glyceraldehyde-3-phosphate dehydrogenase	88.5
*ACT 7*	ppa007211	High	AT5G09810	Actin 7	99.5
*RPL 13*	ppa011512	High	AT3G49010	Breast basic conserved 1	79.7
*TEF 2*	ppa001367	High	AT1G56070	Ribosomal protein S5/Elongation	96.9
*Unknown 8*	ppa011598	High	AT5G50200	nitrate transmembrane transporters	52.5
*AAT 1*	ppa005315	High	AT5G11520	aspartate aminotransferase 3	83.9

Description of candidate housekeeping genes and their expression level according Arismendi et al. [18]

### Evaluation of candidate housekeeping genes for *Prunus* spp. under hypoxia stress

**Fig 1:**
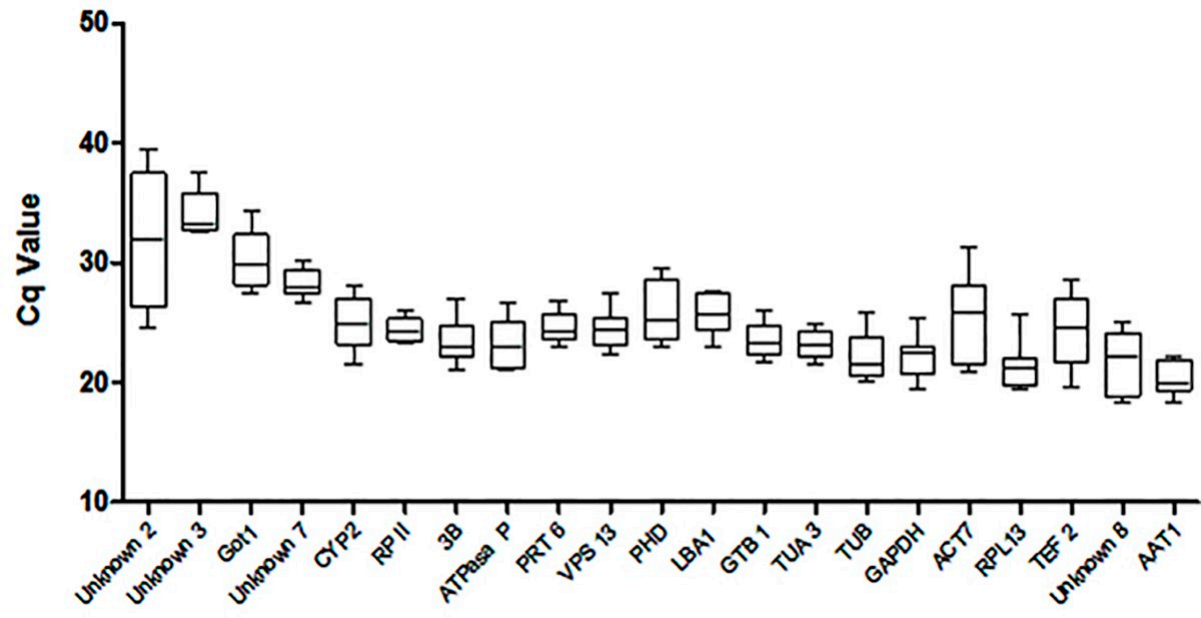
Expression levels of different candidate housekeeping genes for *Prunus* spp. Expression data displayed as RT -qPCR quantification cycle (Cq) values for the housekeeping genes in *Prunus* spp. under hypoxia. The line across the box depicts the median. The box indicates the 25th and 75th percentiles, and whisker caps represent the maximum and minimum values.

The levels of expression of the candidate housekeeping genes were determined by RT-qPCR using root RNA samples from two genotypes of *Prunus* spp. that were subjected to 0 and 72 h of hypoxia stress, while their stability was evaluated with NormFinder software [15]. Table 2 shows primer sequences for the studied housekeeping genes. Quantification cycle values (Cq) of the candidate genes were represented in box and whisker plots (Fig 1) that graphically show gene Cq variation, and thus give good approximations of the best candidate housekeeping genes under these conditions and for these genotypes. Table 3 shows the rankings of candidate housekeeping genes considering overall samples (genotypes and treatments). The stability values ranged between 0.006 (SE±0.002) and 0.061 (SE±0.013). Table 3 also shows rankings that considered genotypes (‘Mariana 2624’ and ‘Mazzard F12/1’) and treatments (0 and 72 h under flooding) separately. There are seven genes highlighted in bold in Table 3 that were selected for further validation analysis because they had higher stability values.

**Table 2:** Primer sequences, RT-qPCR efficiency and amplicon size for each housekeeping gene studied.

Gene name	Forward primer (5’-3’)	Reverse primer (5’-3’)	Tm (°C)	RT-qPCR efficiency	Amplicon expected size (pb)
*Unknown 2*	GTCTGCCAAACACTAACGAACCTATG	AGTGCTTCCAAATGGTGACACAAAT	58.9	2.00	227
*Unknown 3*	TTGACAAACTTGGATGGCAGAACTC	CGAGCATGAGAAATCGTTCCAGTT	58.4	1.97	279
*Got 1*	GTTGGCAGTTGGAAGCACAGC	AAATAAGGGCGCAGACCTCACAT	58.8	2.12	191
*Unknown 7*	AGGCACCTTACTGTGTGGGAGA	GGAACTCCTCTGCGGAACCATT	58.8	2.01	175
*CYP 2*	GGACCTGATTCCAATGGCTCACA	CTTTCCTCATCCCACTGGCTCTG	59.1	2.15	212
*RP II*	TGAAGCATACACCTATGATGATGAAG	CTTTGACAGCACCAGTAGATTCC	56.0	1.96	128
*3B*	CGCACTTACCTCCGTCACCTTC	AAGCCTCCGCCGTCTATACTCA	59.2	1.99	259
*ATPase P*	GTGTCCTCTATGCTCTGTGGCTT	TGCGTCTGCCTCGTGAATGTC	58.9	2.04	182
*PRT 6*	GTGCTGGTGATGACGGTCGTT	TTCTTCGATGCGGATGCCTGTT	59.1	1.87	221
*VPS 13*	CAAGTGTAGCATTCTGGCGTGTG	ATTGGAGGTTGTGACTGTGAGCA	58.7	2.38	189
*PHD*	GGATTCACGGCATGTTCAGCATT	GGAGGCAGAGGCATCATCATCTT	58.6	2.05	247
*LBA 1*	GTAACGACGCCTACACCTTCCTC	CCTGATTATTGCCGCCGCTACT	59.2	1.97	258
*GTB 1*	GTCATCTACGCCGAGTTCACGAA	ACCAGAATCCTGCCTCTCATTGC	59.2	1.99	166
*TUA 3*	TTCTCTCTACTACTCATTCCCTCCTTG	GATTGGTGTATGTTGGTCTCTCG	55.0	1.94	117
*TUB*	CAGGAGAGTGAGCGAGCAGTTC	TCCTCGACGGTAGCATCTTGGTA	59.1	2.07	162
*GAPDH*	TTTGAAGGGTGGAGCGAAGAAGG	ATGGAGTGAACGGTGGTCATCAG	59.0	1.95	210
*ACT 7*	GTTATTCTTCATCGGCGTCTTCG	CTTCACCATTCCAGTTCCATTGTC	56.0	2.07	112
*RPL 13*	GCAGCGACTGAAGACATACAAG	GGTGGCATTAGCAAGTTCCTC	56.0	1.92	103
*TEF 2*	GGTGTGACGATGAAGAGTGATG	TGAAGGAGAGGGAAGGTGAAAG	57.0	2.02	129
*Unknown 8*	GGACAGCCTCAACAAGGACAAGA	CTGACCGTAAGCCACCTCAACAT	59.0	2.03	169
*AAT 1*	GATGGTGGAGAGTGCCTCATAGC	ATACGGTCAGCCATTGCCTTCAA	59.0	2.00	254

**Table 3:** Ranking of putative housekeeping genes according to NormFinder.

Ranking	Genotype & Treatment	Genotype	Treatment
1	***RP II***	***GTB 1***	***TUA 3***
2	***GTB 1***	***Unknown 7***	***Unknown 3***
3	***TUA 3***	***ATPase P***	***PRT 6***
4	*PRT 6*	*PRT 6*	*TUB*
5	*VPS 13*	*TUA 3*	*GTB 1*
6	*Got 1*	*RP II*	*RP II*
7	*LBA 1*	*Got 1*	*RPL 13*
8	*3B*	*3B*	*VPS 13*
9	*GAPDH*	*VPS 13*	*GAPDH*
10	*RPL 13*	*RPL 13*	*LBA 1*
11	*TUB*	*AAT 1*	*Unknown 2*
12	*Unknown 7*	*Unknown 8*	*Got 1*
13	*ATPase P*	*Unknown 3*	*3B*
14	*Unknown 3*	*GAPDH*	*AAT 1*
15	*AAT 1*	*LBA 1*	*Unknown 7*
16	*CYP2*	*TUB*	*ATPase P*
17	*Unknown 8*	*ACT 7*	*CYP 2*
18	*PHD*	*CYP 2*	*TEF 2*
19	*TEF 2*	*PHD*	*PHD*
20	*ACT 7*	*TEF 2*	*Unknown 8*
21	*Unknown 2*	*Unknown 2*	*ACT 7*

Expression stability was calculated by NormFinder algorithms software in roots of ‘Mariana 2624’ and ‘Mazzard F12/1’ under hypoxia considering genotype and treatment.

### Validation of candidate *Prunus* spp. housekeeping genes under new conditions

The previously selected genes, *PRT 6*, *GTB 1*, *ATPase P*, *RP II*, *TUA 3*, *Unknown 3* and *Unknown 7*, were characterized to be validated as housekeeping genes under treatments with additional abiotic stresses, N nutrition and different rootstock genotypes. The evaluation also included two analytical algorithms from BestKeeper [17] and geNorm software [16]. Responses to drought, salinity, cold and heat were evaluated with the hybrid ‘Mariana 2624’ (*P*. *cerasifera* × *P*. *munsoniana*), and to N nutrition with the hybrids ‘Garnem’ [*P*. *dulcis* × (*P*. *persica* × *P*. *davidiana*)] and ‘Colt’ (*P*. *avium* × *P*. *pseudocerasus*).

Table 4 shows the results of drought, salinity, heat and cold stress treatments with the ‘Mariana 2624’ genotype, and N nutrition treatments with the genotypes ‘Garnem’ and ‘Colt’, according to NormFinder software. Stability values ranged from 0.006 (*GTB 1*) to 0.064 (*TUA 3*). According to this analytical algorithm, *GTB 1* is the most stable among treatments and genotypes, followed by *Unknown 7* (Table 4). However, ATPase P was the most stable gene under the ammonium nitrate treatment, followed by the protein-coding genes *PRT 6* and *Unknown 7*. The stability of the seven candidate housekeeping genes was also evaluated with BestKeeper software algorithms (Table 5). The best two candidate housekeeping genes under hypoxia were *RP II* and *TUA 3*, while the best two under drought, salinity, cold and N nutrition conditions were *Unknown 3* and *PRT 6*. Finally, the best two under heat were *Unknown 3* and *GTB 1*.

**Table 4:** Ranking of candidate protein-coding housekeeping genes calculated by NormFinder software.

Rank	Drought	Salinity	Cold	Heat	N treatment	Colt	Garnem	F-12	M2624	All Genotypes
1	*GTB 1* (0.012)	*Unknown 7* (0.007)	*GTB 1* (0.006)	*GTB 1* (0.012)	*ATPase P* (0.09)	*Unknown 7* (0.28)	*ATPase P* (0.23)	*PRT 6* (0.17)	*GTB 1* (0.28)	*GTB 1* (0.33)
2	*Unknown 7* (0.016)	*PRT 6* (0.013)	*Unknown 7* (0.009)	*Unknown 7* (0.017)	*PRT 6* (0.015)	*ATPase P* (0.29)	*PRT 6* (0.30)	*GTB 1* (0.17)	*Unknown 7* (0.61)	*TUA 3* (0.61)
3	*PRT 6* (0.016)	*GTB 1* (0.025)	*TUA 3* (0.010)	*ATPase P* (0.020)	*Unknown 7* (0.016)	*GTB 1* (0.37)	*TUA 3* (0.47)	*TUA 3* (0.21)	*ATPase P* (0.74)	*PRT 6* (0.62)
4	*ATPase P* (0.019)	*ATPase P* (0.027)	*PRT 6* (0.014)	*Unknown 3* (0.024)	*GTB 1* (0.034)	*PRT 6* (0.47)	*Unknown 7* (0.81)	*RP II* (0.67)	*PRT 6* (0.81)	*Unknown 7* (0.65)
5	*Unknown 3* (0.034)	*RP II* (0.029)	*Unknown 3* (0.024)	*PRT 6* (0.026)	*RP II* (0.034)	*RP II* (0.50)	*RP II* (0.99)	*Unknown 3* (1.06)	*TUA 3* (1.02)	*ATPase P* (0.68)
6	*RP II* (0.052)	*TUA 3* (0.033)	*ATPase P* (0.024)	*RP II* (0.029)	*TUA 3* (0.037)	*TUA 3* (0.61)	*GTB 1* (1.23)	*ATPase P* (1.08)	*Unknown 3* (1.19)	*RP II* (0.88)
7	*TUA 3* (0.064)	*Unknown 3* (0.034)	*RP II* (0.063)	*TUA 3* (0.034)	*Unknown 3* (0.086)	*Unknown 3* (2.13)	*Unknown 3* (2.76)	*Unknown 7* (2.44)	*RP II* (1.35)	*Unknown 3* (1.26)

Ranking of candidate protein-coding housekeeping genes under different conditions and using distinct genotypes in order of their expression stability calculated by NormFinder software. Stability value for each condition and gene is show in brackets

**Table 5:** Candidate housekeeping genes ranking considering each treatment and genotype.

Hypoxia			Colt		
Rank	SD (± CP)	CV (%CP)	Rank	SD (± CP)	CV (% CP)
*RP II*	0.74	3.03	*GTB1*	0.66	2.94
*TUA 3*	0.95	4.11	*Unknown 7*	0.71	2.72
*Unknown 7*	**1.01**	3.57	*RPII*	0.71	2.72
*PRT 6*	**1.03**	4.18	*ATPase P*	0.71	3.1
*GBT 1*	**1.03**	4.38	*PRT6*	0.76	3.22
*Unknown 3*	**1.48**	4.36	*TUA 3*	0.77	3.36
*ATPase P*	**1.90**	8.12	*Unknown 3*	**1.38**	4.47
**Drought**			**Garnem**		** **
*Unknown 3*	0.17	0.55	*Unknown 3*	0.16	0.51
*PRT 6*	0.36	1.61	*PRT6*	**1.19**	4.77
*ATPase P*	0.44	1.92	*ATPase P*	**1.50**	6.07
*Unknown 7*	0.53	2.00	*TUA3*	**1.64**	7.05
*GTB 1*	0.74	3.24	*RPII*	**1.73**	6.16
*RP II*	**1.00**	4.03	*Unknown 7*	**1.73**	6.16
*TUA 3*	**1.03**	4.49	*GTB 1*	**1.97**	8.17
**Salinity**			**Mazzard F-12**		** **
*Unknown 3*	0.17	0.55	*RP II*	0.47	0.95
*PRT 6*	0.36	1.61	*PRT6*	0.89	3.67
*ATPase P*	0.44	1.92	*Unknown 7*	**1.00**	3.58
*Unknown 7*	0.53	2.00	*TUA3*	**1.10**	4.75
*GTB 1*	0.74	3.24	*GTB1*	**1.12**	4.80
*RP II*	**1.00**	4.03	*Unknown 3*	**1.60**	4.63
*TUA 3*	**1.03**	4.49	*ATPase P*	**1.70**	7.41
**N treatment**			**Mariana 2624**		** **
*Unknown 3*	0.83	2.64	*Unknown 3*	0.67	2.10
*PRT 6*	0.98	4.03	*GTB1*	0.79	3.48
*GTB 1*	**1.00**	4.38	*TUA 3*	0.88	3.85
*ATPase P*	**1.03**	4.30	*ATPase 3*	0.88	3.86
*TUA 3*	**1.15**	4.97	*Unknown 7*	0.91	3.41
*RP II*	**1.20**	4.45	*PRT 6*	0.98	4.36
*Unknown 7*	**1.20**	4.45	*RP II*	**1.05**	4.20
**Cold**			**Overall**		** **
*Unknown 3*	0.11	0.36	*Unknown 3*	0.85	2.65
*PRT 6*	0.19	0.86	*GTB1*	0.95	4.16
*ATPase P*	0.30	1.34	*Unknown 7*	**1.01**	3.78
*GTB 1*	0.32	1.46	*TUA 3*	**1.01**	4.43
*TUA 3*	0.44	2.03	*ATPase P*	**1.02**	4.42
*Unknown 7*	0.52	1.96	*PRT 6*	**1.24**	5.36
*RP II*	**1.53**	6.07	*RP II*	**1.43**	5.66
**Heat**					
*Unknown 3*	0.16	0.50				
*GTB 1*	0.43	1.95				
*ATPase P*	0.52	2.34				
*PRT 6*	0.54	2.48				
*Unknown 7*	0.55	2.13				
*RP II*	0.60	2.52				
*TUA 3*	0.62	2.75				

The expression stability value was calculated by BestKeeper. Abbreviations: SD [± CP], standard deviation of the CP; CV [%CP], coefficient of variance expressed as a percentage on the CP level; CP, crossing point values. In bold are the genes unsuitable under experimental conditions.

The best housekeeping genes for the genotypes were analyzed with NormFinder and BestKeeper software (Tables 4 and 5). NormFinder software identified different housekeeping genes as the most suitable for each genotype. *GTB 1* was the most suitable gene for the ‘Mariana 2624’ genotype, including the analysis that considered all the genotypes (Table 4). Best Keeper software identified *Unknown 3* as the most suitable for the ‘Mariana 2624’ and ‘Garnem’ genotypes, and as first when the analysis included all the genotypes: ‘Mariana 2624’, ‘Mazzard F12/1’, ‘Colt’ and ‘Garnem’.

**Fig 2:**
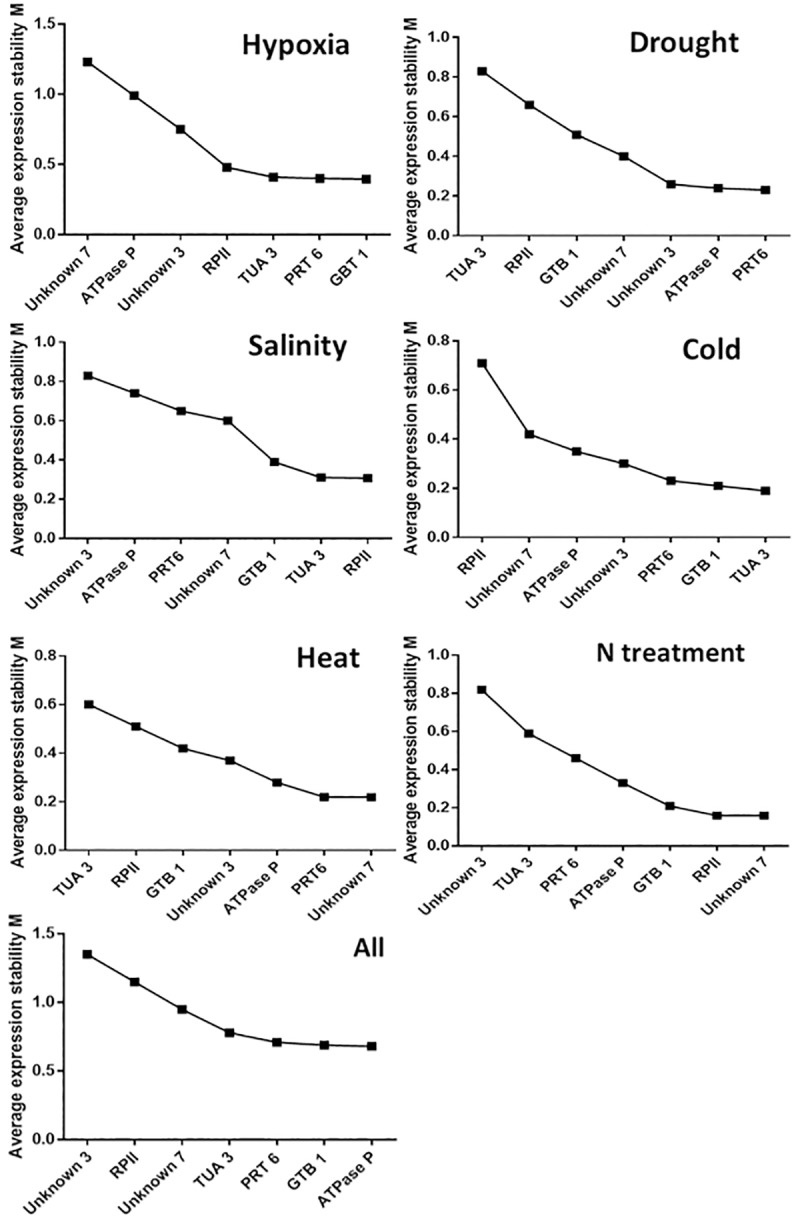
Average expression stability (M value) of the seven selected protein-coding candidate housekeeping genes using geNorm software. Expression stability was evaluated in samples from *Prunus* spp. under drought, salinity, cold, heat, N treatment and all together, plus the results of hypoxia. A lower average expression stability M value indicates more stable expression.

**Fig 3:**
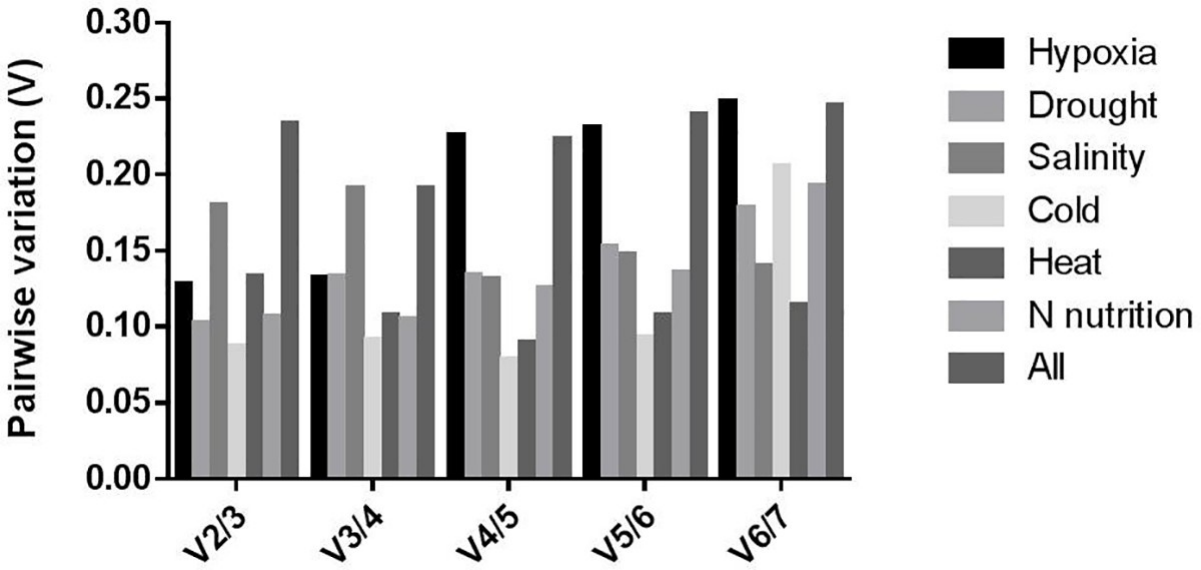
Pairwise variation (V) analysis of the seven candidate housekeeping genes using geNorm software. The pairwise variation (Vn/Vn_+1_) to determine the optimal number of housekeeping genes required for RT-qPCR data normalization. The cutoff value is 0.15.

The consistency of expression of seven candidate housekeeping genes was evaluated with geNorm software [16]. The stress conditions were analyzed separately and then overall treatment conditions and genotypes were analyzed together (Fig 2). The best candidate housekeeping genes are those with expression M-values close to zero. The reference parameters are applicable when the threshold M-value is lower than 1.5 [16]. Most candidate genes had stability M-values lower than 1.5, indicating their suitability as housekeeping genes under these conditions. According to geNorm software, an optimal normalization factor can be calculated as the geometric mean of the best housekeeping genes according to the studied condition (Fig 3). Two housekeeping or reference genes is optimal under hypoxia, drought, cold, heat and N nutrition conditions. The addition of third reference gene does not significantly change the pairwise variation factor (V) However, four housekeeping genes is optimal under salinity with the minimal V-value for this stress (Fig 3). In this case an optimal normalization factor can be calculated as the geometric mean of the housekeeping genes: *RP II*, *TUA 3*, *GBT 1* and *Unknown 7*.

Finally, Tables 4 and 5 and Fig 2 show rankings with all the Cq data obtained with NormFinder, BestKeeper and geNorm. These results include those of the seven selected housekeeping genes considering all treatments and genotypes. NormFinder and geNorm software identified *GBT 1* as the best candidate gene when all conditions and genotypes were evaluated (Table 4, Fig 2), while BestKeeper identified *GBT 1* as the second-best candidate gene (Table 5).

## Discussion

The rootstock for *Prunus* spp. is often from the same subgenus, and even the same species, as the scion, *Amygdalus* (peaches), *Prunus* (plums) and *Cerasus* (cherry), but inter-species and inter-genus hybrids are increasing as the result of efforts to broaden the genetic basis of breeding programs to improve biotic and abiotic resistance/tolerance. Knowledge about the genetic and molecular bases of important rootstock traits is highly desirable in order to reduce breeding periods that can easily exceed 20 years. Identifying and validating genes associated with important traits and responses require knowledge of their expression pattern under different environmental conditions and treatments. The simplest and most popular method to asses gene expression is relative quantification, which requires reference genes to normalize RT-qPCR data. However, the scarcity of validated housekeeping genes for hybrid *Prunus* rootstock species represents a serious limitation.

A housekeeping gene is an internal standard that is assumed to remain constant among experimental groups [11,26]. The expression of housekeeping genes should vary minimally among different tissue and physiological states of the organism [13]. A significant error in estimating the expression of the chosen housekeeping genes increases noise in the assay and makes it impossible to detect small changes. Worse yet, if the expression of the housekeeping gene is altered by the experimental conditions under study, the results may be entirely incorrect. Therefore, it is essential to validate potential housekeeping genes to establish whether they are appropriate for a specific experimental purpose [26]. Studies have shown that the expression patterns of many classical housekeeping genes, such as *Actin*, *β-TUB*, *GAPDH*, and *eEF-1a*, can vary under certain conditions [27,28].

The above mentioned, indicate that existing a real necessity to identify reliable reference genes to strength molecular studies in *Prunus* spp. rootstocks. Microarray and transcriptome databases are excellent resources to search for new candidate housekeeping genes, enabling identify several genes with more stable expression than classical reference genes in some plant species [28–31]. In the present study, 611 candidate housekeeping genes were identified from RNAseq data from our previous work that compared root transcriptomes of two rootstocks ‘Mariana 2426’ and ‘Mazzard F12/1’ under hypoxia treatments (S1 Table). Zhou et al. [31] used more stringent criteria than us to search for housekeeping genes from a RNA-seq data set of apple rootstock, namely fold change values of -0.1 to +0.1, with the same CV <0.3. We identified only 12 genes as housekeeping candidates using Zhou’s criterion (S2 Table). We used a less strict filter because our interest was to capture a broad range of expression of candidate housekeeping genes. Although the number of reads depends directly on the depth of the sequence experiment, the classification of candidate genes according in our study was according to their relative expression. These ranges of gene expression were chosen to find suitable reference genes for better relative quantification of genes of interest with low, intermediate or high levels of expression. Of the 21 candidate genes, 4 had low levels of expression, 10 had intermediate levels, and 7 high levels (Table 1). Eight of the selected candidate genes are protein coders (*ACT*, *CYP2*, *RPII*, *RPL13*, *GAPDH*, *TUB*, *TUA* and *TEF2*) that were recommended by Tong et al. [19], who identified reference genes to study gene expression in different tissue of *P*. *persica* with RT-qPCR. But these genes only represent the categories of intermediate (*CYP 2*, *RP II*, *TUA*) and high levels of expression (*TUB*, *GAPDH*, *ACT*, *RPL 13*, *TEF 2*). Recently, five of the selected candidate gene (*CYP 2*, *RP II*, *TUA*, *TUB*, *RPL 13*) were studied by Klumb et al. [32] as reference genes in tissues of peach (*P*. *persica*) and plum cultivars under flooding.

Based on intra- and inter-group variations within hypoxia treatments and two genotypes (‘Mariana 2426’ and ‘Mazzard F12/1’) determined by Normfinder algorithms, seven of the 21 preselected genes with best stability values were selected for further validation analysis (Table 3). Two of the seven (*RP II*, *TUA 3*) were recommended by Tong et al. [19]. While Klumb et al. [32] described *RP II* as most stable in leaves of ‘Mariana 2624’, but not in roots. In our NormFinder analysis *RP II* was the best ranked in roots considering both treatment (hypoxia) and genotypes (‘Mariana 2624’ and ‘Mazzard F12/1’) (Table 3). For root tissue Klumb et al. [32] recommended *RPL13* as reference gene, which was tenth in our Normfinder ranking (Table 3).

Regarding the roles of the proteins that encoded the housekeeping genes selected in our study, the codified proteins of *Unknown 3* and *Unknown 7* genes have functions that remain undetermined, RP II, RNA polymerase II is an enzyme responsible for catalyzing the transcription of gene-encoding proteins [33]; ATPase P is a protein in the plasma membrane that maintains homeostatic balance [34]; PRT 6, proteolysis 6 is a protein that regulates the destruction of other proteins that are no longer needed by the cell [35]; GTB 1, global transcription factor group B1 is a protein responsible for regulating transcription elongation, related to yeast Spt6 protein, which functions as part of a protein complex in transcription initiation and also plays a role in chromatin structure / assembly [36], and TUA 3, α-tubulin is a globular protein that is part of the microtubule, the main structural component of the cytoskeleton. The TUA protein allows transport through the cell, together with β-tubulin [37] and is one of the most widely and traditionally housekeeping gene used in plant studies, especially in salt, drought, sulfate starvation and ABA experiments [38,39].

The expression stability of these seven genes was evaluated under new treatments and with new genotypes using NormFinder, BestKeeeper and geNorm tools (Tables 4 and 5, Fig 2). The new experimental conditions consisted of subjecting the hybrid genotype ‘Mariana 2624’ to heat, cold, drought and salinity stress, and the hybrids ‘Garnem’ (*P*. *persica* x *P*. *davidiana*) and ‘Colt’ (*P*. *avium* x *P*. *pseudocerasus*) to different doses of ammonium nitrate. The results of validating housekeeping genes generally varied according to the software, treatment and genotype.

According to NormFinder, *GBT1* and *Unknown 7* ranked the highest under drought, cold, and all analyzed conditions, and all genotypes. The best housekeeping gene under salinity was *Unknown 7*, followed by *PRT 6* and *GBT 1*. Finally, the best two candidate protein-coding genes in the nitrogen treatment were *ATPase P* and *PRT 6*.

The genes *Unknown 3* and *PRT 6* ranked as the best housekeeping genes under drought, salinity, cold and N nutrition conditions according to BestKeeper analysis. *PRT 6* was the most often the best housekeeping gene, followed by *Unknown 3* (Table 5). *Unknown 3* was the only suitable housekeeping gene for the ‘Garnem’ genotype, but was also suitable for the ‘Mariana 2624’ genotype, and when all the genotypes and treatment were considered (Table 5).

All genes analyzed by geNorm software under different experimental conditions had stability M-values <1.5, and were suitable as reference genes. This statistical tool predicts the optimal number of housekeeping genes necessary to normalize the experiment. Most treatments analyzed with geNorm software in this study need two reference genes, except under conditions of salinity, where four genes are required for normalization (Fig 3).

High- or middle-ranking genes generally vary slightly, depending on the analytical algorithm used (Tables 4 and 5, Fig 2). In contrast, we observed more coincidence among the poorest performing housekeeping genes under the different conditions. For example, *TUA 3* and *RP II* performed the poorest among the seven genes under drought and heat conditions according the three analytical programs (Tables 4 and 5, Fig 2).

The cut-off value to determine the optimal number of reference genes for RT-qPCR normalization using geNorm software is 0.15. A value under 0.15 indicates that additional reference genes are not required [16]. According geNorm software, no optimal number of reference genes could be determined when all the conditions and genotypes were analyzed together, because variability between sequential normalization factors (based on the n and n+1 least variable reference targets) is relatively high (geNorm V > 0.15). This result concurs with several studies that indicated that different housekeeping genes must validated in accordance with the experimental conditions (Fig 2).

We validated seven housekeeping genes in this study for use in RT-qPCR analysis of *Prunus* spp. roots. Most of these genes had not been described before in *Prunus*, but two (*RP II* and *TUA 3*) were recommended by Tong et al. [19] and Klumb [32]. The expressions levels of seven genes are low (*Unknown 3* and *Unknown 7*) and intermediate (*RP II*, *PRT 6*, *TUA 3*, *ATPase P* and *GTB 1*). None of the genes in this set had high expression levels. They were discarded early from among the 21 preselected genes because of their high Cq levels under hypoxia stress using NormFinder complement (Tables 4 and 5). Therefore, as [40] described, this approach can also validate low and intermediate expression housekeeping genes, in contrast to more “traditional” housekeeping genes whose expression is significantly higher than that of the genes of interest.

It important to clarify that roots are composed of different cellular zones. Three zones have been identified to date along the longitudinal axis of the primary Arabidopsis root: the root apical meristematic zone (RAM) with two domains **[**the proliferative (PD) and the transition domains (TD)**]**, the elongation zone (EZ), and the maturation zone (MZ) [41,42]. These different zones need to be considered when determining suitable reference genes, especially in developmental genetic studies of root morphogenesis. These results will facilitate new genic expression studies of *Prunus* spp. that can improve our understanding of the molecular mechanisms of plants under abiotic or other stress.

This study validated housekeeping *Prunus* spp. genes for normalizing gene expression analysis with RT-qPCR, which can be used in new gene expression studies. Our results suggest that different combinations of suitable housekeeping genes should be selected for normalization according to the genotypes, tissue or treatments to be evaluated.

## Supporting information

S1 Table (XLSX)

S2 Table (XLSX)
